# Design principles for social-ecological research at the landscape scale applied to western Rwanda

**DOI:** 10.1371/journal.pone.0330704

**Published:** 2025-08-22

**Authors:** Matthias Baumann, Dula Duguma, Susanne Vögele, Meike Wollni, Ping Sun, Gaelle Ndayizeye, Joern Fischer

**Affiliations:** 1 Geography Department, Humboldt-University Berlin, Berlin, Germany; 2 Social-Ecological Systems Institute (SESI) Faculty of Sustainability, Leuphana Universität, Lüneburg, Germany; 3 Department of Agricultural Economics and Rural Development, Georg-August Universität Göttingen, Göttingen, Germany; Northeastern University (Shenyang China), CHINA

## Abstract

Place-based social-ecological systems research provides major opportunities to advance sustainability and often involves large, interdisciplinary groups. Researchers adopt various methodologies when studying landscapes, gathering a wide array of data such as socioeconomic information from households, ecological data from specific areas, and qualitative insights from interviews. To integrate these varied methods, we propose identifying social-ecological research units as shared anchor points for data collection across teams. We outline four design principles: (i) spatial scale of social-ecological units, (ii) key social-ecological gradients in the study area, (iii) accessibility of stratification data, and (iv) flexibility in response to logistical challenges. We applied these principles to design a social-ecological study on ecosystem restoration in western Rwanda. We identified five distinct and spatially homogenous clusters, from which we sampled a total of 152 villages (~9.5% of all villages in our study area), which will be visited by different researchers within our study consortium, hence enabling to identify cross-sectional similarities and differences. Through our stratification according to these principles, we created a framework to guide interdisciplinary collaboration. This structured approach supports integration of diverse research efforts and offers insights for advancing place-based social-ecological systems research globally. Sharing our stratification data and methodology, we highlight its potential applicability to other landscapes and sustainability challenges.

## Introduction

Place-based social-ecological systems research and landscape sustainability science are potentially powerful ways to advance sustainability [1,2]. This type of research often is conducted within large interdisciplinary research groups and consortia, all of which address their individual research questions using a variety of methods, and, importantly, across different spatial scales [3]. As a result, the identification of cross-cutting insights – one central goal of large interdisciplinary research teams – can be challenging and hinges on an appropriate sampling design, which may range from the level of housing blocks [4] to the village level. Defining a suitable spatial scale and clearly identifying associated sampling units is therefore important.

In social-ecological research, the landscape scale is especially attractive because it minimizes trade-offs between possible sampling efforts at the local level while still offering the ability to generalize insights across larger spatial extents [5,6]. For example, while higher-resolution entities (such as individual patches and households) can be very idiosyncratic, lower-resolution entities (such as entire nations) may generalize across social and ecological features in ways that mask out important differences (e.g., between ecosystems or communities) [1]. Likewise, landscapes as spatial units are meaningful from both an ecological and a social perspective. Ecologically, the science of landscape ecology has a long and deep history with clearly established ecological principles (e.g., [7–9]), while socially, landscapes are intuitively identified by people as socially and environmentally distinct units [10,11]. Moreover, the social and ecological characteristics of landscapes typically co-evolve [12], making landscapes useful focal points for studying social-ecological interactions and their changes.

Social-ecological research projects at the landscape scale typically rely on data collection at a series of finer-resolution entities, considering different types of entities with different levels of replication. For example, socioeconomic data is often collected at well over one hundred households within a given landscape [13,14], and ecological data typically originates from a similar number of individual patches representing certain land uses within the landscape [15,16]. On the contrary, in-depth interviews about landscape perceptions may come from just tens of people, from few places or even a single community or village within a landscape [17,18]. As such, two challenges commonly arise for social-ecological research projects: (i) diverse types of questions are addressed through data from different specific entities within the landscape; but still, (ii) the integration of the diverse data being generated should ultimately be possible to answer questions about social-ecological relationships or dynamics. One way to address these challenges is to identify social-ecological units from the outset, so that these can be used for various purposes by different researchers, for example for quantitative socioeconomic surveys, for ecological surveys, as well as for in-depth qualitative social science studies [19,20]. Following this logic, an early task in multi- or interdisciplinary projects at the landscape scale therefore is to identify social-ecological units in a structured way, possibly even before all details about specific data collection methods and sample sizes are fully known. Cooking recipes to select such units are rarely available or are often tailored to the conditions of the respective study system or the thematic focus of the research [4]. A selection of guiding principles that are preferably applicable independently of the study context and that can be applied to select sampling units in a structured way, however, is missing.

With this article, we share our approach of selecting social-ecological units at the outset of a large, 4 + years interdisciplinary project (https://ecosystemrestoration.net/), as well as the data and results of the selection process. Our project focuses on western Rwanda and seeks to examine the dynamics and outcomes of ecosystem restoration from a social-ecological perspective (Duguma et al. *in review*). Prospective work within the project will entail (among others) household level socio-ecological and socioeconomic surveys, ecological surveys, and in-depth interviews with local people. Rwanda has pledged to restore large amounts of degraded land, including in its Western Province, the most forested region of the country [21,22]. Until now, most ongoing ecosystem restoration activities have used primarily non-native trees (e.g., *Pinus spp., Eucalyptus spp., Alnus acuminata, Grevillea robusta*) which are generally considered unfavorable for native fauna and biodiversity [23]. Ecologically, restoration is assessed in relation to reference states, including both degraded land as a negative reference state (e.g., cleared hillslopes) and largely intact land as a positive reference state (e.g., in protected areas). Relevant social dynamics and outcomes (e.g., regarding wealth or justice) are likely to be driven by numerous gradients in the landscape, including access to roads and towns, topography, and proximity to strictly protected areas.

Drawing on our experience in western Rwanda, we show a sampling design based on hierarchical clustering that addresses key challenges for social-ecological research projects by following four broad design principles. First, social-ecological units need to be chosen at a scale (or a nested set of scales) that is meaningful both socially and ecologically. Second, data used for the stratification should be accessible, of high quality, preferably open-source data, and somewhat broader in nature than variables collected later in the field to prevent circularity in analyses. Third, the sampling design should be based on a stratification that captures key gradients in socioeconomic and ecological characteristics and should be reproducible in principle [24], without producing bias in data collected after the sampling procedure. Fourth, procedures must be in place to allow for flexibility in case some sites cannot be reached for logistic reasons or are especially important and therefore must not be missed for specific research questions (e.g., via intentionally ‘over-sampling’ villages in remote clusters).

The contribution of this paper is twofold: on the one hand, we provide a robust sample of social-ecological units, which will be used by project members of a large-scale research project and beyond to acquire actual data for western Rwanda. More broadly, we provide a methodological protocol to operationalize the four guiding principles outlined above that could be applied in diverse social-ecological systems worldwide.

## Methods

**Fig 1:**
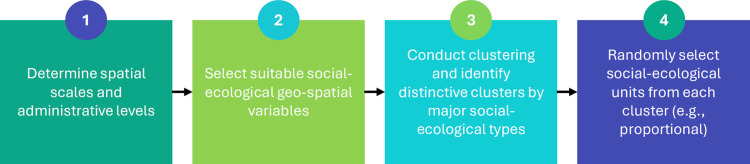
Workflow for the identification of social-ecological units.

To operationalize the design principles for our study area in western Rwanda outlined in the Introduction, we followed four methodological steps (Fig 1). Briefly, we first determined the spatial scales and administrative levels of the social-ecological units to be considered. Second, we described our social-ecological units using a suite of geospatial variables. Third, we applied hierarchical clustering and identified major types of social-ecological system constellations based on environmental factors, land cover and population variables. Fourth, we used stratified random sampling to select social-ecological units from within each cluster.

### Study area

We developed our sampling design in the context of our more than four years lasting research project (https://ecosystemrestoration.net/) in western Rwanda. This project takes a place-based social-ecological systems approach to inform ecosystem restoration. Rwanda is recognized as a global leader in restoration and has pledged to restore two million hectares of land (Duguma et al. *in review*). Historically, agricultural expansion as well as fuelwood harvesting and artisanal mining has led to widespread forest loss [18]. Today, western Rwanda is an agriculture-dominated region, in which approximately two thirds of its households are engaged in crop framing or livestock husbandry [25]. The region’s high population density of more than 650 inhabitants per km^2^ and resulting land scarcity prompted farming even in marginal areas, such as hillslopes [26]. In response to resulting soil erosion, recent and ongoing ecosystem restoration activities have focused on planting fast-growing non-native tree species, including *Pinus spp., Eucalyptus spp. or Alnus acuminata* [27].

### Determining spatial scales and administrative levels

Our first consideration was that the units to be analyzed would need to be meaningful from both an ecological and social perspective. Socially, we reasoned that administrative boundaries would be vital to consider because these guide political decisions, e.g., related to landscape management. Our study area encompasses four official districts; districts are officially subdivided into administrative sectors, administrative sectors subdivided into administrative cells, and administrative cells, in turn, are comprised of several villages. Ecologically, we considered the grain size of the landscape in terms of its topography and land cover gradients. By global comparison, western Rwanda has a relatively fine spatial grain, with individual field sizes as well as patches of trees often measuring less than half a hectare; and distances from hilltops to valleys of often only a few hundred meters. Drawing on this information, we decided to focus on the administrative cell level for the initial stratification because broad landscape features (such as distance from towns or amount of tree cover) were captured well at this scale, and we considered that these were also meaningful from a governance perspective. However, we considered that actual data collection on the ground would likely focus on smaller areas – e.g., particular patches of trees, or the households within a given village. For that reason, our stratification encompassed an initial stratification at the cell level; but then a stratified random selection of villages within cells, which would be targeted for data collection. We note that a different choice of scales would have been possible, and perhaps no less appropriate – our purpose here is not to suggest that other approaches would have been wrong, but to make the logic of our decisions transparent.

### Data preparation

We characterized all administrative cells within our study area using freely available geospatial variables describing topography (i.e., slope, elevation, topographic wetness index), infrastructure and population (i.e., distance to road, distance to major streams, number of houses per km^2^, distance to major towns), major land cover types (i.e., woodlands, pastures, cropland, etc.), and other important contextual variables (i.e., distance to major lakes, distance to National Park land) (Table 1). We included these variables for the clustering, as they are commonly used to identify archetypical social-ecological systems [28,29]. We refrained from including other variables including, for example, census data or national data on poverty or health, as collecting such data during field interviews represents one major component of the larger research unit. We z-transformed all variables (mean: 0, standard deviation: 1), before calculating a correlation matrix to check for correlation between potential candidate variables. We considered two variables being correlated to each other for r> 0.5 and iteratively removed variables such that (i) we no longer had strong correlations between the variables, while (ii) maintaining a maximum number of meaningful variables for clustering (Fig 3A), and to meaningfully describe the individual clusters afterwards. While removing correlated variables may not be strictly necessary for the clustering technique we applied, it resulted in a smaller number of more easily interpretable variables that capture essentially the same variance in the underlying data – thereby contributing to parsimony and interpretability.

**Table 1:** Geospatial variables summarized at the cell level (i.e., NUTS-4).

Variable group	Extracted variable	Source
Topography	1. Mean elevation 2. Mean slope 3. Mean Topographic Wetness Index 4. Distance to major streams	1. ASTER digital elevation model [30] 2. ASTER digital elevation model [30] 3. [31] 4. OpenStreetMaps
Infrastructure & population	1. Distance to major roads 2. Housing density (houses/km^2^) 3. Distance to major towns	1. OpenStreetMaps 2. Google Open Buildings [32] 3. OpenStreetMaps
Other contextual variables	1. Distance to National Park 2. Distance to major lakes	1. World Database on Protected Areas [33] 2. OpenStreetMaps
Major land cover types	1. % Forest 2. % Arable land 3. % Pasture 4. % Shrubland	1-4: ESA World Cover 2020 [34]

**Fig 2:**
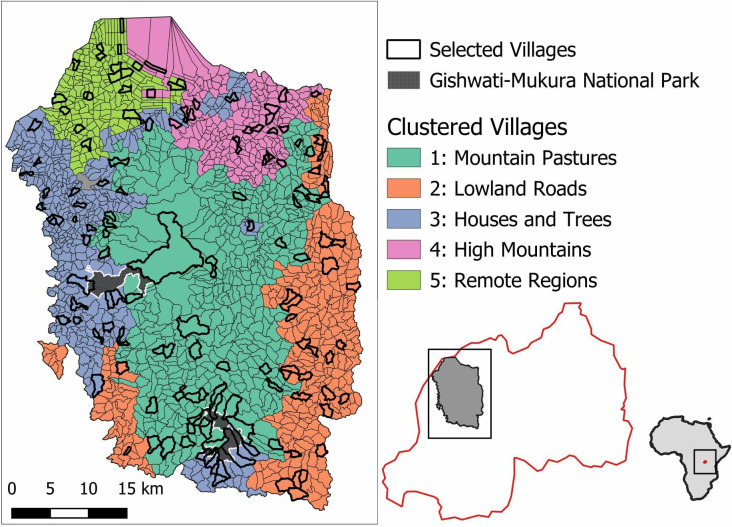
Results of the clustering analysis. The large map shows the villages colored according to their cluster. The small maps show the location of the study area inside Rwanda and within Africa. The maps were created using freely available data from https://gadm.org and RStudio [43].

**Fig 3:**
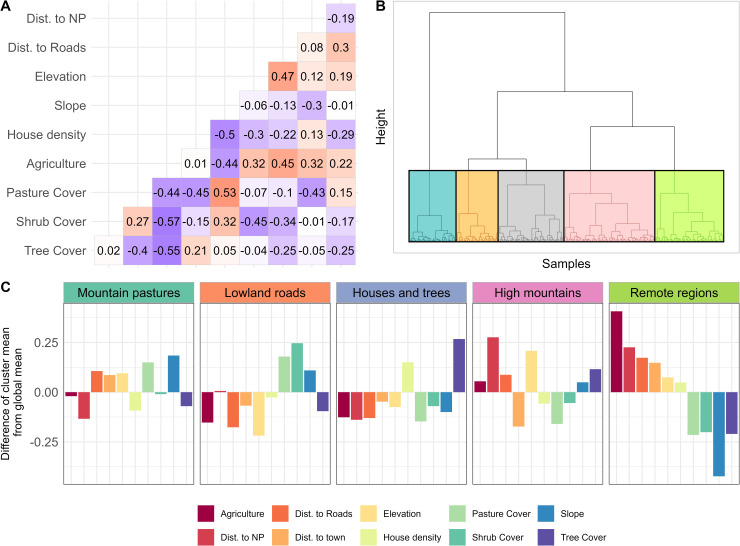
(A) Correlation matrix of the variables used in the clustering after checking for collinearities; for better representation, we omitted the labels of the x-axis which are the same as on the y-axis (B) Dendrogram from the hierarchical clustering and identification of five distinct clusters; (C) Differences in cluster means from global means for each of the variables used for hierarchical clustering.

### Hierarchical clustering of cells

Drawing on the final set of sufficiently uncorrelated geospatial variables, we applied hierarchical clustering to identify major types of administrative cells across our study region. Specifically, we applied divisive hierarchical clustering, which has been previously used to cluster spatial units in social-ecological systems [29]. Hierarchical clustering splits at each level a group of observations into two clusters based on a criterium of heterogeneity. We used Ward’s minimum variance method to group observations into clusters. Ward’s method minimizes the within-cluster variance and hence produces relatively compact clusters, which served our purpose well. We screened the dendrogram of administrative cells as well as resulting cluster maps to identify the optimal number of clusters qualitatively, ensuring a more or less homogenous distance between clusters on one hand, and coherence in geographical space of the clusters on the other (Fig 2). Finally, we summarized the distinctive characteristics of each cluster by calculating for each input variable the difference between the mean value within each cluster and the global mean value (i.e., across all clusters).

### Selection of social-ecological units

Following the delineation of clusters of administrative cells, we randomly selected *villages* (i.e., NUTS-5 units) from each cluster proportional to the respective cluster size (Table 1). We set the minimum number of villages to be selected per cluster at 20 to ensure sufficient observations for analyses within clusters, resulting in a total sample size of 152 villages. In addition, for the two largest clusters containing national park land (Cluster 1 and Cluster 3), we ensured that both included a minimum number of 10 villages immediately adjacent to one of the two national parks. This step of purposive ‘over-sampling’ was taken to ensure that locations immediately adjacent to the national parks were included in the final selection of villages – for some of the social science questions, the inclusion of enough villages immediately adjacent to the national parks was a high priority that would not have been captured by a completely random stratification. Next, within each cluster, we randomly assigned ranks *[1, n]* to each village. These ranks represent the priority with which the villages in any given cluster should be visited during field visits. The ranking ensures that researchers collecting different kinds of data (e.g., household data, ecological data, interview data) have an overlap in the villages they visit, regardless of their total sample size. Such an overlap will, in turn, facilitate data integration later in the project.

## Results

Through hierarchical clustering, we identified five clusters of varying size. The smallest cluster includes 220 villages (representing 13.4% of all villages in our study area), whereas the largest cluster includes 476 villages (29.0%, Table 2, Fig 2). Stratifying by cluster, we randomly selected a total of 152 villages that serve as social-ecological units in our research context (Table 2).

**Table 2:** Cluster sizes (i.e., number of villages within a selected cluster) and the total number of villages selected from each cluster based on a stratified random sampling (for more detail, please refer to the methods). The selected villages will serve as social-ecological units within a larger research consortium, allowing for the identification of cross-cutting insights between different sub-projects.

#	# Villages in cluster	% Villages in cluster relative to all villages	# of selected villages
1	476	29.0%	44
2	331	20.2%	31
3	397	24.2%	37
4	217	13.2%	20
5	220	13.4%	20

Cluster 1 (“*Mountain pastures”*) encompasses the central region of our four study districts and is characterized by a mountain ridge running from north to south. This cluster is distinguished by its steep slopes, setting it apart from the surrounding clusters. It contains a high amount of pastureland with relatively sparse tree cover. Additionally, the housing density in this cluster is notably lower than in the other four clusters, correlating with its greater distance from main roads and towns. Because this cluster borders roughly half of the area of the two national park patches, the mean distance to these parks is shorter than that of most other clusters. These characteristics point to prioritizing soil stabilization and reforestation in areas of steep slopes and sparse tree cover. In addition, the proximity to two national parks may offer opportunities for buffer zone restoration and corridor creation to enhance connectivity (Fig 3).

Cluster 2 (*“Lowland roads”*) primarily stretches along the eastern edge of the plateau, with a smaller portion extending to its southwestern edge. It is the lowest of all five clusters in elevation but the closest to main roads, which are predominantly situated around the high plateau area. Among the five clusters, cluster two has the highest shrubland cover, a land cover class that includes extensive banana plantations. Like cluster one, this cluster also contains substantial amounts of pastureland, highlighting potentials for agroecological production in the context of restoration projects. Additionally, cluster two contains less arable land compared to most of the other clusters (Fig 3).

Cluster 3 (*“Houses and trees”*) is located on the opposite side of cluster two, extending predominantly along the western edge of the study area adjacent to Lake Kivu and encompassing a portion of Gishwati Forest. A smaller segment of this cluster occurs in the southern part of the study area, bordering Mukura Forest. The cluster features the highest population density, with a shorter distance to main roads and towns. Additionally, tree cover within this cluster is relatively high, while pasture and shrublands are less prevalent. Like cluster two, arable land cover in cluster three is relatively less than in the central and northern clusters. With regards to restoration activities, focus should be on home gardens due to limited availability of space for large-scale restoration projects (Fig 3).

Clusters four and five are situated in the northern part of the study area, with cluster four occupying the northwest and cluster five the northeast. Both clusters are far from Gishwati-Mokura national park patches and have less pastureland than the central and eastern clusters (one and two). Despite their proximity, there are notable differences between the two northern clusters. Cluster 4 (“*High mountains*”) includes especially high elevations, likely suffering widespread soil erosion. This cluster borders the major road in the north, is relatively close to major population centers and has high levels of high tree cover. Contrary, cluster 5 (*“Remote regions”*) features the flattest terrain with moderate elevation and has the lowest tree cover among all clusters but the highest proportion of arable land. The remoteness may hint towards higher success rates of decentralized and community-led activities (Fig 3).

## Discussion

With a growing number of multi- and interdisciplinary investigations of entire landscapes – often framed as social-ecological systems research or landscape sustainability science – developing ways to systematically select social-ecological units for diverse sampling purposes is vital [35]. Here, we provided an example of a robust and pragmatic sampling design to undertake place-based social-ecological systems research in western Rwanda, following a series of broad guiding principles. We discuss our findings in the context of (i) trade-offs between design principles; (ii) representation of social-ecological diversity within the units ultimately selected, and (iii) the possible application of the same or a similar stratification process in other social-ecological settings and contexts.

### Trade-offs between design principles

While we followed a clear logic for data selection and stratification, we obviously faced trade-offs regarding our four principles. For example, while all our data for stratification was open-source data that would be easily acquirable in other study areas as well (principle 2), gradients in socio-economic factors (e.g., differences in household income) could only be approximated through spatial variables (i.e., distance to major town, distance to roads). Furthermore, socio-cultural factors (principle 3), such as ethnographic distribution, attitudes, values, or beliefs [36], were not explicitly considered during the clustering. Missing such factors can be interpreted as limitation, as they are certainly important factors to capture key gradients in social characteristics (e.g., data on poverty, health, household income etc.). However, we emphasize that landscape-scale spatial representations of these types of variables are absent for many regions in the world and therefore difficult to consider in sampling design *a priori*. When such data are not used (as in our case), a good alternative can be to identify useful proxies or correlates that can be considered in the stratification exercise. In our case, we included agricultural practices (e.g., farmers vs. pastoralists), which are likely to correlate with some of the socio-cultural variables, at a spatial scale fine enough to highlight differences between regions (principle 1). Lastly, our approach ‘over-sampled’ locations surrounding Gishwati-Mukura National Park to address individual research priorities within the consortium. While this may introduce some spatial bias, we weighed the value of statistical independence higher, which we ensured by maintaining a minimum number of villages per cluster (principle 4). While these trade-offs between design principles imply that our approach has some limitations, they also demonstrate the flexibility and adaptability of the approach. Overall, this highlights that our approach – despite its limitations – is useful for selecting representative sampling units that are suitable for different research interests within a large multi-disciplinary research consortium.

### Representation of social-ecological diversity within sampling units

Social-ecological units are relatively homogenous units of similar characteristics [35,37,38], even though there is no guarantee that the units are necessarily very different from their neighbors [35]. Our approach addresses this uncertainty by employing a multi-level strategy. Specifically, we identified clusters (i.e., social-ecologically homogenous regions) at the coarser NUTS-4 level (i.e., *cells*), thus ensuring that broad-scale characteristics were grouped across western Rwanda. By selecting our final social-ecological sampling units at the finer NUTS-5 level (i.e., *villages*) we seek to capture the remaining social-ecological diversity within clusters. We propose that this multi-level approach – together with the guiding principles – provides a consistent way for combining multiple ways of data collection and analysis [29,39] in diverse social-ecological systems. Our approach thus offers an innovative and broadly transferable sampling strategy allowing for cross-cutting research – which is a central goal of large interdisciplinary research projects. In addition, the approach shown here represents the starting point of a 4 + year long research project, in which social-ecological aspects of restoration in western Rwanda are being examined from different angles, for example by means of extensive interviews and participatory Geographic Information Systems (PPGIS). Data gathered in these sub-projects are fully independent from the data used here for our sampling design and hence offer opportunities to *validate* the clusters and their characteristics discussed in this paper a posteriori.

### Application to other social-ecological settings

We also suggest that our approach of identifying a set of representative social-ecological system units in relatively data-scarce parts of the Global South can be readily adapted to other social-ecological settings. While the broad principles and general approach we used should be transferable, we note that the number and type of variables used, landscape characteristics such as landscape structure and land-use intensity [9], spatial grain and extent of the landscape [40], and scalar issues across time, space, and disciplines [36] will need to be adjusted carefully for any new study system. For example, where better datasets (e.g., more accurate land cover data) are available, these can easily replace globally available datasets. Likewise, the selection of the spatial units for clustering and sampling may differ depending on the overall goal of the larger research project. Different specific choices may be appropriate, for example, when planning to generate land-use change scenarios for integrated coastal management [41] or to guide landscape planning [35] when answering specific questions around qualitative topics such as equity dimensions of protected area management [42]. As such, the most important transferable aspect of our work is the general approach and set of guiding principles – we argue that by *a priori* formulating a set of guiding principles, the structured definition and identification of social-ecological sampling units can be guided in any landscape around the world.

## Supporting information

S1 Data baumann-etal_data. (ZIP)

S1 File baumann-etal_Rwanda_SES-Sampling_script. (HTML)
