# Supplementary material for: Design principles for social-ecological research at the landscape scale applied to western Rwanda
Source: PLoS One. 2025 Aug 22;20(8):e0330704. doi: 10.1371/journal.pone.0330704 (PMC12373253; doi:10.1371/journal.pone.0330704)
Supplement: S1 File — (HTML) [file pone.0330704.s002.html]

Baumann et al. Design principles for social-ecological research at the landscape scale and their application to western Rwanda


# Baumann *et al.* Design principles for social-ecological research at the landscape scale and their application to western Rwanda

### Code documentation

This script documents the process described in the manuscript “Design
principles for social-ecological research at the landscape scale and
their application to western Rwanda”. All data tables are used here are
visualized in form of the first 5 rows to demonstrate the shape of the
data. The original dataset will be made available upon request.

## Load packages and data

```
library(readxl)
library(tidyverse)
library(ggcorrplot)
library(sf)
library(RColorBrewer)
library(ggh4x)
library(dendextend)
library(ggdendro)
```

```
# Change this folder when running this on your own machine
workFolder <- "D:/ResearchArchive/Publications-accepted/2025/baumann_Rwanda-Sampling/v09_Proofing-after-acceptance/"
variables <- read_excel(paste0(workFolder,"baumann-etal_inputVariables.xlsx"), sheet = "Area_LULC_adm4")

head(variables, 5)
```

```
## # A tibble: 5 × 36
##   DistrictID District  SectorID Sector CellID Cell     Perc_TC Perc_Shrb Perc_PS
##        <dbl> <chr>        <dbl> <chr>   <dbl> <chr>      <dbl>     <dbl>   <dbl>
## 1         22 Ngororero      301 Bwira    1540 Bungwe     12.8       9.79    30.8
## 2         22 Ngororero      301 Bwira    1541 Cyahafi    11.8      10.1     45.6
## 3         22 Ngororero      301 Bwira    1542 Gashubi    16.3       5.91    27.7
## 4         22 Ngororero      301 Bwira    1543 Kabaron…   13.1       6.32    38.6
## 5         22 Ngororero      301 Bwira    1544 Ruhinda…    8.70     11.7     35.0
## # ℹ 27 more variables: Perc_AL <dbl>, Perc_house <dbl>, MEAN_slope <dbl>,
## #   MEAN_twi <dbl>, MEAN_elv <dbl>, MEAN_road <dbl>, MEAN_river <dbl>,
## #   MEAN_parks <dbl>, MEAN_riverStream <dbl>, MEAN_town <dbl>, MEAN_pH <dbl>,
## #   MEAN_OC <dbl>, MEAN_clay <dbl>, MEAN_PatchCoh <dbl>, MEAN_Wvg <dbl>,
## #   var_Wvg <dbl>, var_Patch <dbl>, var_Slope <dbl>, var_twi <dbl>,
## #   var_elv <dbl>, var_road <dbl>, var_town <dbl>, var_pH <dbl>, var_OC <dbl>,
## #   var_Clay <dbl>, Mean_lake <dbl>, Soil_BN <dbl>
```

## Check for correlation between clustering co-variates

The steps taken here are: 1. Subset `variables` by
selecting columns used for the analysis. Rename the columns to match
names used in the manuscript. 2. Build a correlation matrix. Examine
this matrix to identify variables with high correlation (r>0.7) to
other variables. These variables will be excluded from the analysis. 3.
Filter variables based on the correlation matrix and the specific
characteristics of the study area. Specifically we filter
`Perc_house < 0.1` (i.e., to remove cells with larger
urban areas) and `MEAN_lake> 2000m` (i.e., to account for
the particular characteristics of the cells near the shoreline of Lake
Kivu).

```
# Create a vector of variables that we take from the input file
varSelect <- c("CellID","Perc_TC", "Perc_Shrb", "Perc_PS", "Perc_AL", "Perc_house", "MEAN_slope", "MEAN_twi", "MEAN_elv", "MEAN_road", "MEAN_river", "MEAN_parks", "MEAN_town", "Mean_lake", "Soil_BN")
```

```
# Subset the datafile based on the vector above, and create a correlation matrix
var_sub <- variables %>% select(all_of(varSelect)) %>% filter(Perc_house < 0.1 & Mean_lake > 2000)
```

```
# Rename the variables
var_sub <- var_sub %>%
  rename(
    "Tree Cover" = "Perc_TC",
    "Shrub Cover"   = "Perc_Shrb",
    "Pasture Cover" = "Perc_PS",
    "Agriculture" = "Perc_AL",
    "House density" = "Perc_house",
    "Slope" = "MEAN_slope",
    "TWI" = "MEAN_twi",
    "Elevation" = "MEAN_elv",
    "Dist. to Roads" = "MEAN_road",
    "Dist. to River" = "MEAN_river",
    "Dist. to NP" = "MEAN_parks",
    "Dist. to town" = "MEAN_town",
    "Dist. to Lake Kivu" = "Mean_lake",
    "Soil" = "Soil_BN"
  )

head(var_sub)
```

```
## # A tibble: 6 × 15
##   CellID `Tree Cover` `Shrub Cover` `Pasture Cover` Agriculture `House density`
##    <dbl>        <dbl>         <dbl>           <dbl>       <dbl>           <dbl>
## 1   1540        12.8           9.79            30.8        43.8         0.0153 
## 2   1541        11.8          10.1             45.6        32.0         0.00885
## 3   1542        16.3           5.91            27.7        48.9         0.0120 
## 4   1543        13.1           6.32            38.6        40.7         0.0110 
## 5   1544         8.70         11.7             35.0        41.7         0.0149 
## 6   1545        16.0          25.6             35.6        18.0         0.0166 
## # ℹ 9 more variables: Slope <dbl>, TWI <dbl>, Elevation <dbl>,
## #   `Dist. to Roads` <dbl>, `Dist. to River` <dbl>, `Dist. to NP` <dbl>,
## #   `Dist. to town` <dbl>, `Dist. to Lake Kivu` <dbl>, Soil <dbl>
```

```
var_sub %>% select(-CellID) %>% cor() %>% round(2) %>% ggcorrplot(lab=TRUE, type="lower", lab_size = 2)
```

The following variables were removed: `Dist. to River`,
`TWI`, `Soil`,
`Dist. to Lake Kivu`.

```
var_final <- var_sub %>% select(-c("CellID", "Dist. to River", "TWI", "Soil", "Dist. to Lake Kivu"))
var_final %>% cor() %>% round(2) %>% ggcorrplot(lab=TRUE, type="lower", show.legend=TRUE, lab_size = 2)
```

## Apply Hierarchical clustering

Using the dataset, we apply hierarchical clustering to identify
clusters of similar cells. The steps taken in this section are: 1.
Standardize the dataset. See here for
more information. 2. Calculate the distance matrix using the Manhattan
method. 3. Apply hierarchical clustering using the Ward method. 4. Add
the cluster label to the dataset.

The visualization below already colors the final decision on the
cluster number. For a discussion on how the decision was taken, please
refer to the main manuscript.

```
#Standardization
cluster_data.est<-scale(var_final)
#Distance matrix
dist_matrix<-dist(cluster_data.est, method="manhattan")
#Cluster analysis
cluster<-hclust(dist_matrix, method="ward.D")
# Make the cut
cut <- cutree(cluster, 5)
```

```
dend <- as.dendrogram(cluster)
dend_data <- ggdendro::dendro_data(dend)

ggplot(segment(dend_data)) +
  geom_segment(aes(x = x, y = y, xend = xend, yend = yend), linewidth=0.2) +
  theme_light() +
  labs(x = "Samples", y = "Height") + 
  theme(axis.title.y = element_text(size=12),
        axis.title.x = element_text(size=12),
        axis.text.y = element_blank(),
        axis.text.x = element_blank(),
        legend.position = "none",
        axis.ticks = element_blank(),
        panel.grid.minor = element_blank(),
        panel.grid.major = element_blank()) +
  geom_rect(aes(xmin = 0, xmax = 38.5, ymin = 0, ymax = 115), fill = "#66c2a5", alpha = 0.01, color = "black") +
  geom_rect(aes(xmin = 38.5, xmax = 73, ymin = 0, ymax = 115), fill = "#fc8d62", alpha = 0.01, color = "black") +
  geom_rect(aes(xmin = 73, xmax = 127, ymin = 0, ymax = 115), fill = "#8da0cb", alpha = 0.01, color = "black")  +
  geom_rect(aes(xmin = 127, xmax = 201.5, ymin = 0, ymax = 115), fill = "#e78ac3", alpha = 0.01, color = "black") +
  geom_rect(aes(xmin = 201.5, xmax = 258, ymin = 0, ymax = 115), fill = "#a6d854", alpha = 0.01, color = "black")
```

```
# Add the cluster label to the original dataset
ds_label <- var_sub %>% mutate(Cluster=cut)
```

## Build visualizations for assessing variables

In this section we visually examine the output from the clustering.
Specifically, we 1. Map the cluster label to a regional shapefile and
visualize the clusters. 2. We calculate the cluster means of the
clustering variables and compare them to the global mean of the
variables.

### Map visualization

```
shp <- st_read(paste0(workFolder,"RWA_adm4_UTM.shp"), quiet = TRUE)
shpVals <- shp %>% left_join(ds_label, by = join_by(ID_4 == CellID)) %>% filter(Cluster %in% c(1,2,3,4,5))

ggplot() +
  geom_sf(data = shpVals, aes(fill = as.factor(Cluster))) +
  theme_minimal() +
  theme(legend.title=element_blank()) + 
  ggtitle("Villages assigned to clusters", subtitle = "The map was created using freely available data from https://gadm.org")
```

### Assess global and cluster means

Calculate differences between global mean and cluster mean for each
variable. First, standardize the variables.

```
# Scale the variables between 0 and 1
ds_scaled <- ds_label %>% select((-c("Dist. to River", "TWI", "Soil", "Dist. to Lake Kivu"))) %>% 
  mutate_at(vars(-c("CellID", "Cluster")), function(x){(x-min(x))/(max(x)-min(x))}) %>% 
  select(-"CellID") %>% pivot_longer(!c(Cluster), names_to="Variable", values_to="value")

# Calculate global and cluster mean of each variable
globalMeans <- ds_scaled %>% group_by(Variable) %>% summarise(Mean = mean(value))
clusterMean <- ds_scaled %>% group_by(Cluster, Variable) %>% summarise(Mean = mean(value))

# Merge them together, calculate the differences
all <- merge(clusterMean, globalMeans, by.x="Variable", by.y="Variable") %>% rename(ClusterMean=Mean.x) %>% rename(globalMean=Mean.y) %>% mutate(Diff = ClusterMean - globalMean)
```

```
# Define the colors for the facet boxes
map_colors <- c("0" = "#000000",  # Light grey for background
                      "1" = "#66c2a5",  # Custom colors for clusters
                      "2" = "#fc8d62",
                      "3" = "#8da0cb",
                      "4" = "#e78ac3",
                      "5" = "#a6d854")
cluster_colors <- setNames(map_colors, c("0", "1", "2", "3", "4", "5"))

get_cluster_color <- function(cluster) {as.character(map_colors[as.character(cluster)])}

facet_colors_only <- as.vector(sapply(levels(factor(all$Cluster)), get_cluster_color))

cluster_names <- c("1" = "Mountain pastures", "2" = "Lowland roads", "3" = "Houses and trees", 
                   "4" = "High mountains", "5" = "Remote regions")
```

```
library(ggh4x)
library(RColorBrewer)
# Create a plot
ggplot(all, aes(x=Variable, y=Diff, fill=Variable)) +
  geom_bar(stat='identity') +
  scale_fill_brewer(palette = "Spectral") +
  theme_bw() +
  labs(y="Difference of cluster mean\n from global mean") +
  guides(fill = guide_legend(nrow = 2)) +
  theme(legend.position = "bottom",
        legend.title=element_blank(),
        axis.text.x=element_blank(),
        axis.text.y=element_text(size=12),
        axis.ticks=element_blank(),
        axis.title.x=element_blank(),
        strip.text = element_text(size = 12, color = "black"),
        strip.background = element_rect(color = "darkgrey")) +
   facet_wrap2(~ Cluster, strip = strip_themed(background_x = elem_list_rect(fill=facet_colors_only, alpha = 0.001)), labeller = as_labeller(cluster_names), nrow = 1)
```

## Select villages

In this last step we select villages inside the clusters. For details
on the process please refer to the main manuscript. Steps taken here: 1.
Load a a second shapefile containing the villages (i.e., NUTS-5 level).
The file already contains information whether or not a village is
intersecting with Gishwati Mokura National Park. Load also a shapefile
of the Gishwati-Mokura National Park for visualization 2. Determine the
sample size and the number of villages sampled around Gishwati Mokura
National Park 3. Randomly select villages based on the rules described
in the manuscript and point 2. Apply `set.seed(42)` for
reproducability.

```
# Load village file and join clustering output
adm5 <- st_read(paste0(workFolder, "RWA_adm5_UTM.shp"), quiet = TRUE) %>% 
  select(c("NAME_2", "NAME_3", "NAME_4", "NAME_5", "ID_4", "ID_5", "Park_YN")) %>% 
  left_join(ds_label[,c("CellID", "Cluster")], by=join_by(ID_4 == CellID)) %>% filter(Cluster %in% c(1,2,3,4,5))

# Load the national park shapefile
parks <- st_read(paste0(workFolder,"parks.shp"), quiet = TRUE)
```

```
set.seed(42)
```

```
# Determine
smallestSize <- 20
# Calculate the size of each group
clusterSizes <- adm5 %>% count(Cluster) %>% mutate(perc = n/sum(n))
# Identify the smallest cluster, assign minimal sample size
clusterSizes <- clusterSizes %>% mutate(sampleSize = ifelse(n==min(n), smallestSize, 0))
# Assign the other ones proportionally
clusterSizes <- clusterSizes %>% mutate(sampleSize = round(ifelse(sampleSize != smallestSize, perc*smallestSize / min(perc), sampleSize)))

head(clusterSizes)
```

```
## Simple feature collection with 5 features and 4 fields
## Geometry type: GEOMETRY
## Dimension:     XY
## Bounding box:  xmin: 751007.5 ymin: 9769206 xmax: 797626.7 ymax: 9833379
## Projected CRS: WGS 84 / UTM zone 35S
##   Cluster   n                       geometry      perc sampleSize
## 1       1 476 POLYGON ((769109.7 9778789,... 0.2900670         44
## 2       2 331 MULTIPOLYGON (((768477.3 97... 0.2017063         31
## 3       3 397 MULTIPOLYGON (((756616.7 98... 0.2419257         37
## 4       4 217 POLYGON ((776441.3 9816086,... 0.1322364         20
## 5       5 220 POLYGON ((759597.9 9813499,... 0.1340646         20
```

```
# Cluster 1 --> 10 around the NP, and 34 from the rest
c1_a = adm5 %>% select("NAME_4", "ID_4", "NAME_5", "ID_5", "Cluster", "Park_YN") %>% 
  filter(Cluster==1 & Park_YN==1) %>% 
  sample_n(size=10) %>% 
  mutate(id = seq(1,10, 1)) %>% 
  select(-Park_YN)
c1_b = adm5 %>% select("NAME_4", "ID_4", "NAME_5", "ID_5", "Cluster", "Park_YN") %>% 
  filter(Cluster==1 & Park_YN==0) %>% 
  sample_n(size=as.numeric(clusterSizes[1,"sampleSize"])[1] - 10) %>% 
  mutate(id = seq(10+1,as.numeric(clusterSizes[1,"sampleSize"])[1],1)) %>% 
  select(-Park_YN)

# Cluster 2
c2 = adm5 %>% select("NAME_4", "ID_4", "NAME_5", "ID_5", "Cluster") %>% 
  filter(Cluster==2) %>% 
  sample_n(size=as.numeric(clusterSizes[2,"sampleSize"])[1]) %>% 
  mutate(id = 1:as.numeric(clusterSizes[2,"sampleSize"])[1])

# Cluster 3 --> 10 around the NP, and 27 from the rest
c3_a = adm5 %>% select("NAME_4", "ID_4", "NAME_5", "ID_5", "Cluster", "Park_YN") %>% 
  filter(Cluster==3 & Park_YN==1) %>% 
  sample_n(size=10) %>% 
  mutate(id = seq(1,10, 1)) %>% 
  select(-Park_YN)
c3_b = adm5 %>% select("NAME_4", "ID_4", "NAME_5", "ID_5", "Cluster", "Park_YN") %>% 
  filter(Cluster==3 & Park_YN==0) %>% 
  sample_n(size=as.numeric(clusterSizes[3,"sampleSize"])[1] - 10) %>% 
  mutate(id = seq(10+1,as.numeric(clusterSizes[3,"sampleSize"])[1],1)) %>% 
  select(-Park_YN)

# Cluster 4
c4 = adm5 %>% select("NAME_4", "ID_4", "NAME_5", "ID_5", "Cluster") %>% 
  filter(Cluster==4) %>% 
  sample_n(size=as.numeric(clusterSizes[4,"sampleSize"])[1]) %>% 
  mutate(id = 1:as.numeric(clusterSizes[4,"sampleSize"])[1])

# Cluster 5
c5 = adm5 %>% select("NAME_4", "ID_4", "NAME_5", "ID_5", "Cluster") %>% 
  filter(Cluster==5) %>% 
  sample_n(size=as.numeric(clusterSizes[5,"sampleSize"])[1]) %>% 
  mutate(id = 1:as.numeric(clusterSizes[5,"sampleSize"])[1])

fullSample <- rbind(c1_a, c1_b, c2, c3_a, c3_b, c4, c5)
```

## Visualize final sample

Visualize the selected villages inside the study area. Show the table
of the selected villages.

```
ggplot() +
  geom_sf(data = adm5, aes(fill = as.factor(Cluster))) +
  geom_sf(data = parks, fill="black") +
  geom_sf(data = fullSample, color="black", fill=NA) +
  theme_minimal() +
  theme(legend.title=element_blank()) +
  ggtitle("sampled villages", subtitle = "The map was created using freely available data from https://gadm.org")
```

```
#sf::st_drop_geometry(fullSample)
fullSample %>% rename(Cell = NAME_4, CellID = ID_4, Village = NAME_5, VillageID = ID_5) %>% sf::st_drop_geometry()
```

```
##              Cell CellID          Village VillageID Cluster id
## 1         Ruronde   1994           Gisozi     13647       1  1
## 2          Kageyo   1959           Ntonde     13361       1  2
## 3        Bugarula   1580          Gatomvu     10787       1  3
## 4          Gihira   1986          Bitenga     13588       1  4
## 5          Kageyo   1959           Mucaca     13360       1  5
## 6         Kibanda   1596           Ruhuha     10881       1  6
## 7         Ruronde   1994      Nyamibombwe     13651       1  7
## 8         Ruronde   1994           Kigali     13648       1  8
## 9          Kageyo   1959 Site Mukura ya 1     13365       1  9
## 10         Kageyo   1959          Rukondo     13364       1 10
## 11        Tangabo   1956           Rugano     13339       1 11
## 12         Tetero   1574          Mizingo     10759       1 12
## 13     Nyabipfura   1612       Nyirabwina     10982       1 13
## 14         Mashya   1583      Karuhindura     10802       1 14
## 15       Runyinya   1556          Marembo     10658       1 15
## 16         Busuku   1980           Gatare     13530       1 16
## 17        Karambo   1961          Gituntu     13376       1 17
## 18        Karambo   1961           Dehero     13373       1 18
## 19         Rugeyo   1965          Karambo     13414       1 19
## 20      Kintarure   1683         Kabuguzo     11443       1 20
## 21         Remera   1993          Shyembe     13646       1 21
## 22         Gitega   1676          Cyanika     11381       1 22
## 23          Ngoma   1983           Gisayo     13560       1 23
## 24         Mashya   1583           Kagano     10801       1 24
## 25         Bungwe   1540           Rutoyi     10576       1 25
## 26         Kavumu   1630          Munyege     11100       1 26
## 27          Ngoma   1983         Cyeshero     13558       1 27
## 28         Muyira   1955        Rutangaza     13331       1 28
## 29         Kageyo   1959           Nyanzu     13362       1 29
## 30       Rwantobo   1662          Rurembo     11282       1 30
## 31        Karambo   1786      Ndongoshori     12261       1 31
## 32      Kintarure   1683           Mabare     11444       1 32
## 33        Busunzu   1557           Gitaba     10662       1 33
## 34        Musenyi   1611         Gisakavu     10975       1 34
## 35      Kintarure   1683         Munanira     11445       1 35
## 36          Gitwa   1977           Mubuga     13506       1 36
## 37         Busuku   1980           Busuku     13526       1 37
## 38         Kabona   1991         Rusheshi     13625       1 38
## 39        Kibanda   1596         Kimirehe     10879       1 39
## 40          Mwana   1679           Murama     11411       1 40
## 41        Muramba   1794          Tubindi     12308       1 41
## 42         Kagano   1609         Gitabage     10963       1 42
## 43          Mberi   1992           Kagano     13634       1 43
## 44          Mberi   1992           Gakeri     13628       1 44
## 45         Rususa   1602           Cyumba     10918       2  1
## 46        Cyahafi   1541          Rushubi     10580       2  2
## 47          Nsibo   1606       Nyarusange     10947       2  3
## 48        Bambiro   1604       Rwasankuba     10936       2  4
## 49     Nyagahondo   1671          Kabyaza     11343       2  5
## 50      Bweramana   1587           Gasave     10827       2  6
## 51     Cyimanzovu   1681          Murikwa     11435       2  7
## 52        Rugogwe   1590          Murambi     10843       2  8
## 53        Cyahafi   1541           Kamina     10579       2  9
## 54     Cyimanzovu   1681          Bihembe     11431       2 10
## 55         Mugano   1600       Nyabisindu     10907       2 11
## 56          Sanza   1592       Gashyushya     10851       2 12
## 57        Ruhanga   1549           Kamina     10626       2 13
## 58      Ruhindage   1544         Kabirizi     10591       2 14
## 59        Karambo   1548            Nteko     10619       2 15
## 60         Matare   1577             Gako     10772       2 16
## 61          Nsibo   1606          Zegenya     10949       2 17
## 62        Rwamiko   1579           Butare     10783       2 18
## 63       Bitabage   1594        Nyamugari     10870       2 19
## 64        Nyamata   1567          Kibanda     10725       2 20
## 65    Kanyamitana   1682      Kazirankara     11438       2 21
## 66        Ruhanga   1549           Butare     10621       2 22
## 67          Nsibo   1606         Cyambogo     10942       2 23
## 68        Gatsibo   1546          Gatongo     10602       2 24
## 69         Gaseke   1605        Ngobagoba     10941       2 25
## 70     Nyagahondo   1671          Musenyi     11346       2 26
## 71         Mubuga   1588          Burengo     10831       2 27
## 72      Cyarusera   1976           Kunini     13499       2 28
## 73          Gitwa   1570           Biraro     10736       2 29
## 74        Rwamiko   1579       Nyakibande     10784       2 30
## 75        Shyembe   1941          Shyembe     13224       2 31
## 76        Rundoyi   1990         Rushasho     13618       3  1
## 77         Kagano   1958      Cyabatsinga     13344       3  2
## 78        Rundoyi   1990       Rugaragara     13617       3  3
## 79         Kagano   1958            Tumba     13355       3  4
## 80         Mwendo   1962           Kagogo     13385       3  5
## 81    Nyagahinika   1945        Nyarusuku     13252       3  6
## 82         Mwendo   1962         Kagombwa     13386       3  7
## 83         Mwendo   1962           Rugari     13392       3  8
## 84         Kagano   1958          Kamonyi     13348       3  9
## 85         Kagano   1958         Rugomero     13354       3 10
## 86      Terimbere   1984          Kanombe     13570       3 11
## 87         Gisiza   1968          Gihinga     13430       3 12
## 88           Gisa   1831       Kabashanja     12530       3 13
## 89     Rukaragata   1946        Rwamiyaga     13266       3 14
## 90       Busigari   1770         Kanembwe     12150       3 15
## 91         Mwendo   1962            Nyove     13391       3 16
## 92      Terimbere   1984          Gihinga     13568       3 17
## 93         Kinigi   1812            Byima     12400       3 18
## 94      Terimbere   1984          Kasonga     13572       3 19
## 95         Gatovu   1817          Ruhanga     12443       3 20
## 96         Kinigi   1812          Nyamiko     12404       3 21
## 97       Kamuhoza   1785         Kagarama     12253       3 22
## 98         Rugasa   1989         Kabitovu     13609       3 23
## 99         Kabaya   1559           Bitare     10675       3 24
## 100       Mukondo   1820           Busogo     12458       3 25
## 101          Gisa   1831           Gihira     12528       3 26
## 102        Rurara   1974           Gasoro     13478       3 27
## 103        Biruyi   1971         Kabakiza     13457       3 28
## 104        Rubaya   1653        Kaburende     11225       3 29
## 105        Kavumu   1987           Gasasa     13596       3 30
## 106       Bunyoni   1947           Bureke     13268       3 31
## 107        Gatare   1985          Kirinja     13584       3 32
## 108       Bunyoni   1947            Gitwa     13271       3 33
## 109        Nyundo   1821        Nyakagezi     12474       3 34
## 110          Rega   1619         Ngangare     11031       3 35
## 111        Murara   1828           Kabere     12513       3 36
## 112   Bukinanyana   1620           Kibaya     11038       3 37
## 113     Karengera   1643         Mashyuza     11176       4  1
## 114      Kabatezi   1622        Gitambuko     11047       4  2
## 115        Ryinyo   1649           Humiro     11206       4  3
## 116       Basumba   1615             Vuga     11002       4  4
## 117       Gahondo   1675           Kanama     11374       4  5
## 118        Busoro   1638         Gatagara     11138       4  6
## 119       Murambi   1678          Gahondo     11396       4  7
## 120       Rurembo   1673         Cyasenge     11358       4  8
## 121         Rwaza   1680           Murama     11421       4  9
## 122        Ryinyo   1649      Kadaterurwa     11208       4 10
## 123         Rwaza   1680         Musekera     11422       4 11
## 124   Nyirakigugu   1624      Nyamutukura     11064       4 12
## 125     Marangara   1670           Tetero     11340       4 13
## 126       Kintobo   1645          Kansesa     11189       4 14
## 127         Rwaza   1680      Rugarambiro     11427       4 15
## 128      Rugarama   1637         Kinkware     11133       4 16
## 129     Nyagisozi   1646           Dehero     11190       4 17
## 130         Rwaza   1680          Gifunzo     11416       4 18
## 131     Karengera   1643        Rwumuyaga     11179       4 19
## 132      Kabatezi   1622           Kagaga     11048       4 20
## 133       Buringo   1756           Butaka     12053       5  1
## 134      Bihungwe   1799         Bihungwe     12321       5  2
## 135  Nyarushyamba   1809           Kivumu     12377       5  3
## 136       Mirindi   1802          Kiryoha     12339       5  4
## 137          Rega   1625             Rega     11070       5  5
## 138        Rusura   1769           Rebero     12147       5  6
## 139     Ryabizige   1777      Kanyamagare     12181       5  7
## 140        Butaka   1757       Gaheriheri     12060       5  8
## 141      Ndoranyi   1803           Gitega     12344       5  9
## 142        Mutovu   1760          Rindiro     12085       5 10
## 143       Kabumba   1759           Gatovu     12075       5 11
## 144    Rwanzekuma   1776       Rukorakore     12179       5 12
## 145        Gasiza   1764      Nyarunembwe     12117       5 13
## 146         Myuga   1635            Myuga     11125       5 14
## 147        Gasiza   1764           Kibavu     12111       5 15
## 148       Kageshi   1766           Ruhara     12126       5 16
## 149        Mutovu   1760       Bigaragara     12080       5 17
## 150       Mirindi   1802           Tamira     12341       5 18
## 151          Rega   1625        Terimbere     11072       5 19
## 152 Kanyirabigogo   1792          Murambi     12301       5 20
```
